# Prevalence of multidrug-resistant organisms colonizing neonates at a tertiary hospital in Johannesburg, South Africa

**DOI:** 10.1093/tropej/fmaf051

**Published:** 2026-01-02

**Authors:** Nonkululeko Mntla, Vindana Chibabhai, Trusha Nana

**Affiliations:** Clinical Microbiology and Infectious Diseases, University of the Witwatersrand, Johannesburg, 2193, South Africa; Charlotte Maxeke Johannesburg Academic Hospital, National Health Laboratory Services, Johannesburg, 2193, South Africa; Clinical Microbiology and Infectious Diseases, University of the Witwatersrand, Johannesburg, 2193, South Africa; Charlotte Maxeke Johannesburg Academic Hospital, National Health Laboratory Services, Johannesburg, 2193, South Africa; Clinical Microbiology and Infectious Diseases, University of the Witwatersrand, Johannesburg, 2193, South Africa; Charlotte Maxeke Johannesburg Academic Hospital, National Health Laboratory Services, Johannesburg, 2193, South Africa

**Keywords:** neonate, colonization, multidrug resistance, Klebsiella pneumoniae, Acinetobacter baumannii, beta-lactamase, carbapenem resistant, MRSA, Candida auris, Southern Africa

## Abstract

Neonatal mortality remains a global health challenge, particularly in sub-Saharan Africa, where infections often caused by multidrug-resistant (MDR) organisms are a leading cause of death. This study aimed to assess the prevalence of MDR ESKAPE pathogens and *Candida auris* colonization among hospitalized neonates in a non-outbreak setting, identify associated risk factors, and characterize antimicrobial resistance patterns. A cross-sectional sub-study was conducted at a tertiary hospital in South Africa between November and December 2020. A total of 258 rectal and skin swabs were collected from 86 neonates and cultured for ESKAPE organisms and *C. auris*. Isolated MDR organisms underwent further characterization. Of the 135 ESKAPE + *C. auris* isolates identified, 70.4% (95/135) were MDR. Colonization with ESBL-producing *Klebsiella pneumoniae* was most common (65%, 56/86), followed by XDR *Acinetobacter baumannii*. NDM-producing *A. baumannii* (5.8%) was more frequently detected than carbapenemase-producing Enterobacterales (3.9%). A prolonged hospital stay (median 14 days, *P* < .001) was significantly associated with MDR colonization. Rectal and skin swabs provided comparable yields for Gram-negative MDR organisms. The high prevalence of MDR ESKAPE + *C. auris* colonization highlights the value of routine, non-invasive screening for surveillance in neonatal units. Enhanced infection control strategies and improved surveillance systems incorporating colonization swabs and clinical risk profiling are urgently needed.

## Introduction

Neonatal mortality remains a great contributor to global under-5 mortality, with infection accounting for a third of these deaths [1, 2]. The highest burden remains in low- to middle-income countries (LMICs), particularly in sub-Saharan Africa, where timely diagnosis and treatment of neonatal infections are often a challenge [3–6]. As a result, prevention of infection has become important.

The relationship between perinatal colonization and early onset neonatal sepsis (EOS, infection occurring in the first 72 h of life) is well established, and forms the basis of screening and vaccination strategies [7, 8]. In contrast, late onset neonatal sepsis (LOS, infection after 72 h of life), particularly among hospitalized neonates, is more synonymous with healthcare-associated infections (HAI), and thus guides likely etiology and appropriate empiric antimicrobials [3, 5–8].

LOS is largely driven by multidrug-resistant organisms (MDROs) (organisms showing non-susceptibility to one or more agents in three antibiotic classes) among LMICs [3, 5, 7, 9–11]. South African national surveillance performed in public hospitals on culture-confirmed neonatal infections (Baby-GERMS-SA) reports an increase in neonatal infections from 2014 to 2019, particularly Gram-negative sepsis. In fact, LOS incidence in South Africa was 4.9 per 1000 live births, over four times higher than that of EOS [5].

The “ESKAPE” pathogens, namely *Enterococcus faecium*, *Staphylococcus aureus*, *Klebsiella pneumoniae*, *Acinetobacter baumannii complex*, *Pseudomonas aeruginosa*, and *Enterobacter* species, along with *Candida auris*, are of particular concern [12]. These organisms are featured among the WHO priority pathogen list due to their multidrug-resistant (MDR) phenotypes that cause HAIs [13–15]. These include vancomycin-resistant *E. faecium* (VRE), methicillin-resistant *S. aureus* (MRSA), carbapenem-resistant *K. pneumoniae* and *Enterobacter* species [denoted as carbapenem-resistant Enterobacterales (CRE) or carbapenemase-producing Enterobacterales (CPE) and their extended spectrum beta-lactamase-producing (ESBL) phenotypes]. They also include multi-, extensively, and pan-drug resistance among *A. baumannii* and *P. aeruginosa.*

Neonates are vulnerable to invasive infections due to immature skin and mucosal barriers that allow for transient colonization and subsequent opportunistic infection [7, 13, 16–18]. Colonization with MDROs often exceeds rates of invasive infection, and while a direct correlation between colonization and subsequent infection has not always been established, colonization surveillance has proven valuable for outbreak control and transmission prevention [7, 13, 16–22]. Consequently, neonatal units have used screening swabs for MDROs in an attempt to prevent horizontal transmission and outbreaks [17, 18, 21].

This study aimed to determine the prevalence and risk factors associated with MDRO colonization, and to characterize MDR ESKAPE organisms and *C. auris* among neonates admitted to South Africa’s largest tertiary hospital. This aimed to address a significant knowledge gap on MDRO colonization rates in a resource-constrained and often overcrowded neonatal unit, and to identify risk factors that could aid in early outbreak identification and infection control guidelines.

## Methodology

This study is a sub-study of a larger, externally funded research project, which was conducted concurrently and is referred to as the primary investigation (Supplementary material).

### Study design

A cross-sectional survey was conducted in two neonatal intensive care units (ICUs) and two non-intensive care neonatal wards at a public tertiary-level hospital in the country, in which ∼8000 cesarean section deliveries and 19 000 live births occur annually [23]. Sampling and data collection took place between 11 and 18 November 2020 in the ICU, which has a bed occupancy of 45 beds, and between 13 and 15 December 2020 in the non-ICU of the neonatal unit (non-ICU), which has a bed occupancy of 96 beds.

### Patients

The study population included neonates present in the ward on the day of sample collection. The desired sample size was 94 (90 ± 5). For this study, a neonate was defined as an infant who was 28 days old or younger on the day of admission to the unit. Neonates were stratified into two groups based on chronological age to distinguish EOS and LOS (i.e. 72 h).

Neonates were excluded from the study if they had congenital malformations such as imperforate anus, choanal atresia, or cleft palate (to avoid a traumatic sample collection), if they were transferred from other hospitals within 48 h of sampling, if they had gastroschisis or had undergone surgical intervention, and if they were nasally intubated, as this precluded nasal sampling.

### Sample and data collection

Sample collection was done daily in the morning prior to the neonates being bathed. Three screening swabs were collected simultaneously from neonates in every alternate bed according to the standard operating procedures (SOP) (Supplementary material). These included one rectal swab, one skin swab (umbilicus, both axillae, and the groin area sampled with the same swab), and one nasal swab, each labeled with a unique patient number. Each neonate was swabbed once. All swabs were transported to the processing laboratory in Cary Blair transport medium at ±4°C. Data collected from patient medical files included antimicrobial therapy and duration of hospitalization before sampling.

### Laboratory analysis

The following variables were assessed: organism identified, pattern of multidrug resistance, and phenotypic determinants of carbapenem resistance in *K. pneumoniae*, *E. cloacae*, and *A. baumannii.* The screening swabs were placed into Brain Heart Infusion enrichment media (Diagnostic Media Products, National Health Laboratory Service, South Africa) and incubated aerobically overnight at 35°C. Following 16–18 h of incubation, 10 μl of each turbid sample was subcultured onto differential and selective agar. These were reviewed following 16–18-h incubation for growth, and a presumptive identification was made using phenotypic methods, e.g. using colony morphology on selective agar and rapid biochemical tests performed as per SOP (Supplemental material).

If the presumptive identification suggested an ESKAPE + *C. auris* organism, identification was confirmed using the VITEK^®^-MS MALDI-TOF system (bioMérieux, v3.2, Marcy-l’Étoile, France) or the VITEK^®^ 2 system (bioMérieux, Marcy l‘Étoile, France). Antimicrobial susceptibility testing was performed on confirmed ESKAPE + *C. auris* organisms on the VITEK^®^ 2 or by Kirby Bauer disk diffusion. This was interpreted according to Clinical and Laboratory Standards Institute (CLSI) M100 guidelines, and MDROs were categorized as ESBL, MRSA, or CRE/CPE based on CLSI definitions [24]. The definitions for MDR and XDR definitions for *P. aeruginosa* and *A. baumannii* were based on those outlined by Magiorakos *et al*. [11]. All *C. auris* isolated were presumed to be azole resistant and were categorized as MDR. This was based on previously published local data that showed that clade 3 *C. auris* strains, the most isolated and associated with colonization in this region, possessed the azole resistance conferring ERG11 gene mutation [25, 26].

Further characterization included determining the presence of carbapenemases in CRE and *A. baumannii* organisms, using a lateral flow assay (RESIST-4.O.K.N.V., Coris) which tests for OXA-48 (oxacillinase-48)-like, KPC (*Klebsiella pneumoniae* carbapenemase), NDM (New-Delhi metallo-betalactamase-1) and VIM (Verona integron-encoded metallo-betalactamase) carbapenemases. Susceptibility testing was also performed with a vancomycin gradient diffusion strip (Etest^®^)(bioMérieux, Marcy l‘Étoile, France) for MRSA, amphotericin B and micafungin Etests for *C. auris*, and colistin broth microdilution for XDR *A. baumannii.*

### Statistical analysis

Data were compiled and analyzed with Microsoft Excel (Microsoft Corporation, 2019) and Graphpad (Software version 9.3.1, GraphPad Software, Inc, La Jolla, USA). Descriptive statistical analysis was used to analyze the data. Categorical data were recorded as proportions of the overall data in frequencies and percentages. Comparison between categorical variables was performed using Fisher’s exact test. Continuous variables were reported as median and interquartile range and analyzed using the Mann–Whitney *U* test.

Each neonate was included once for the enumeration of MDROs, despite some being colonized with the same pathogen at different body sites. Each ESKAPE + *C. auris* organism isolated was included in the data analysis. Neonates were divided into two groups: Group 1: Neonates colonized with MDR ESKAPE + *C. auris* and Group 2: Neonates with no growth on their swabs, non-ESKAPE + *C. auris* colonized and neonates with non-MDR ESKAPE colonization (i.e. susceptible organisms). To identify risk factors for MDRO colonization, neonatal characteristics were described for each patient, and the following factors were analyzed: the length of hospitalization (days) before the day of sampling, postnatal antimicrobial exposure prior to sampling and the day of life on which sampling was conducted. A two-tailed *P*-value of <.05 was statistically significant.

### Ethical consideration

This sub-study received approval from the Human Research Ethics Committee (Medical) of the University of the Witwatersrand [clearance certificate (approval) number M200583]. The primary investigation received ethical approval from the same committee (M191129).

## Results

Eighty-six neonates were sampled, 43 in the ICU and 43 in the general neonatal wards (non-ICU), and a total of 258 swabs were collected. An equal number of rectal, skin, and nasal swabs were collected from which significant and non-significant (normal skin flora) colonizers were isolated.

**Figure 1:**
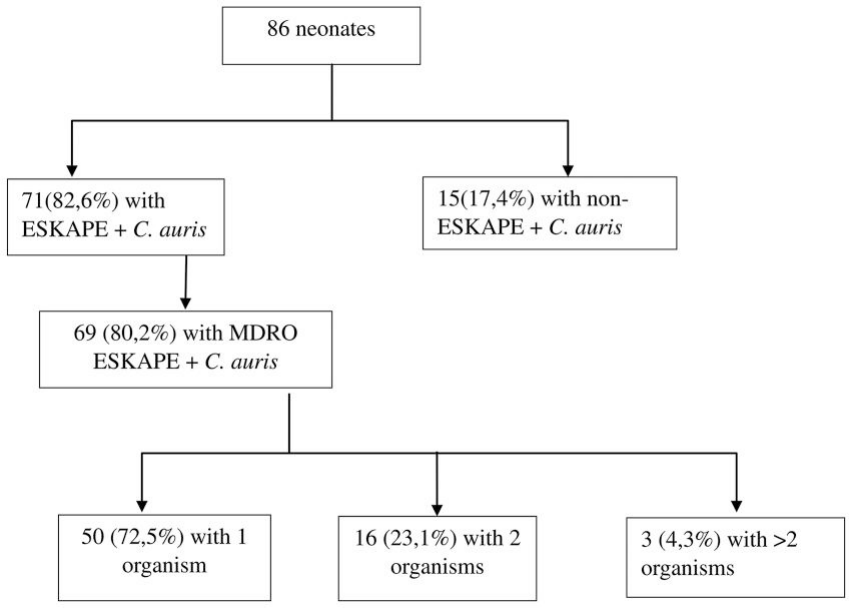
Prevalence of ESKAPE + *C. auris* colonization among neonates.

**Figure 2:**
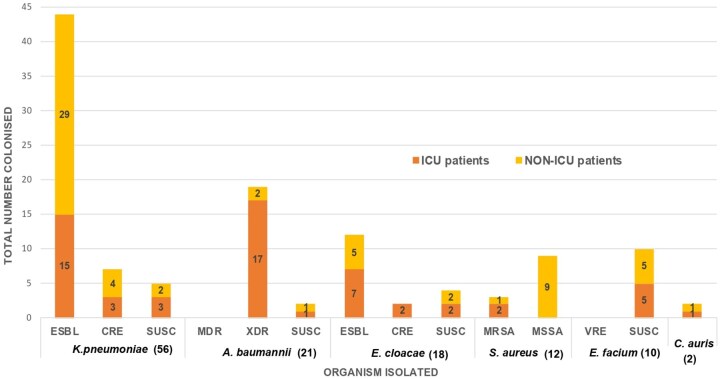
Cumulative prevalence of ESKAPE + *C. auris* organisms among neonates stratified by site (ICU VS NON-ICU). CANAU, *C. auris*; SUSC, Susceptible.

A total of 135 ESKAPE + *C. auris* organisms were isolated from the 258 swabs collected. Sixty organisms were ESBL-producing Enterobacterales, and nine were CREs (of which two tested positive for OXA-48 carbapenemase production). In contrast, among carbapenem-resistant *A. baumannii* (CRAB) isolated, 5/19 (26%), tested positive for NDM carbapenemase production. Figures 1 and 2 display the prevalence of MDR ESKAPE + *C. auris* organisms identified among neonates (Figs 1 and 2). ESKAPE + *C. auris* organisms’ prevalence is discussed in Table 1, and their susceptibility profiles are discussed in Table 2.

**Table 1.** Prevalence of ESKAPE + *C. auris* organisms among swabs collected

	*n* (%) of total organisms isolated: 135
**MDR ESKAPE + *C. auris***	95 (70.4)
MRSA	3 (2.2)
*C. auris*	2 (1.5)
ESBL *K. pneumoniae* and *E. cloacae*	60 (44.4)
CRE/CPE *K. pneumoniae* and *E. cloacae*	9 (6.7)
MDR *A. baumannii*	2 (1.5)
XDR *A. baumannii*	19 (14.1)
Colistin-resistant *A. baumannii* (CRAB)	3/19 (15.8)
**Susceptible bacteria**	40 (29.6)

CPE, carbepenemase-producing Enterobacterales; CRE: carbapenem-resistant Enterobacterales; ESBL, extended spectrum beta-lactamase; MDR, multidrug resistant; MRSA, methicillin-resistant *Staphylococcus aureus*; XDR, extensively drug resistant.

**Table 2.** Cumulative antimicrobial susceptibility report—antimicrobial agents listed by class

										% Susceptibility to routinely used antimicrobials for empiric therapy											
	Organisms	Total number of strains	% of total isolates						Carbapenamases detected	Phenotype	β-lactams					Aminoglycosides		Colistin (BMD) (MIC ≤2)^a^	Vancomycin (MIC ≤4)^a^	Amphotericin B (MIC ≤1)^b^	Micafungin (MIC ≤2)^b^
			Wild type (WT)	ESBL isolates (%)	CRE isolates (%)	MDR (%)	XDR (%)	MRSA (%)			Ampicillin	Cloxacillin	Pip-taz	Cefotaxime	Meropenem	Getamicin	Amikacin				
**GNB**																					
1	*Klebsiella pneumoniae*	64	9	48	7	–			OXA 48+v (*n *= 2*)*	ESBL	IR	–	17	4	100	0	100	–	–	–	–
				75%	11%					CRE	IR	–	0	0	29	29	86	–	–	–	–
										WT	–	100	100	100	100	100	–	–	–	–	–
2	*Acinetobacter baumannii complex*	27	6	–		2	19	–	NDM (*n *= 5)	MDR	–	–	0	IR	50	50	100	–	–	–	–
						7.4%	70.4%			XDR	–	–	0	IR	26	26	47	84	–	–	–
										WT	–	–	100	IR	100	83	100	–	–	–	–
3	*Enterobacter cloacae complex*	19	5	12	2	–			Nil	ESBL	IR	–	23	0	100	46	100	–	–	–	–
				63.2%	10.5%					CRE	IR	–	0	0	0	0	100	–	–	–	–
										WT	IR	–	31	100	100	100	100	–	–	–	–
**GPC**																					
4	*Staphylococcus aureus*	13	10	–				3	–	MRSA	IR	–	–	–	–	33	–	–	100	–	–
								23.1%		WT	IR	100	–	–	–	100	–	–	100	–	–
5	*Enterococcus faecium*	10	10	(No MDROs isolated)					–	WT	10	–	–	IR	–	–	–	–	100	–	–
**Yeast**																					
6	*C. auris*	2	–							All isolates	–									100	100

a Interpretation according to CLSI [24].

b Interpretation according the CDC tentative break points [25].

Wild type (WT): Microorganisms without phenotypically expressed acquired resistance. –: Not routinely tested for in this organism; CPE, carbepenemase-producing Enterobacterales; CRE, carbapenem-resistant Enterobacterales; ESBL, extended spectrum beta-lactamase; IR, intrinsically resistant; MDR, multidrug resistant; MDRO, multidrug-resistant organism; MRSA, methicillin-resistant *Staphylococcus aureus*; “n” represents the number of neonates for whom data was available and for whom statistical analysis was performed; Pip-taz, piperacillin-tazobactam; XDR, extensively drug resistant.

Neonates with MDRO colonization were hospitalized longer than their non-MDRO colonized counterparts (14 vs 2.5 days, *P*-value < .001), and a comparison of MDRO colonized vs non-colonized patients in each section (i.e. ICU vs non-ICU) revealed no increased risk for colonization in ICU regardless of hospitalization period. This is depicted by a median length of hospitalization for MDRO colonized vs non-MDRO colonized (14 vs 6 days in ICU) and (14 vs 2 days in non-ICU) (*P*-value = .3). The neonatal characteristics assessed for association with MDRO colonization are summarized in Table 3.

**Table 3.** Characteristics associated with MDRO colonization

Neonatal characteristics	MDRO colonized (*n* = 67)	Non-MDRO colonized (*n* = 16)	*P*-value
**Days of life at time of sampling**			
Median (IQR)	16 (6–28)	3 (1–6.5)	**.0001^a^**
≤3 days of life	6	9	
>3 days of life	61	7	
**Hospitalization period**			
Median (IQR)	14 (5–25)	2,5 (1–6.5)	**.0001^a^**
≤3days	6	9	
>3days	61	7	
**ICU admission** ^b^	34/69 (49.3%)	9/17 (52.9%)	.99
**Overall postnatal antimicrobial exposure**	52	13	.99
**No antimicrobial exposure**	15	3	
**Beta-lactams**	48	13	.58
Ampicillin	30	8	.8
Carbapenems	13	5	.3
Cephalosporins	1	0	.99
Piperacillin-tazobactam	13	0	.06
**Aminoglycosides**	39	7	.4
**Colistin**	6	0	.6
**Vancomycin**	4	6	**.0028^a^**
**Fluconazole**	4	1	.99
**Amphotericin B**	4	0	.99
**Micafungin**	0	0	–

a Figures in bold represent statistical significance.

b Study numbers were assigned according to the area in which the neonate was hospitalized during sample collection, therefore “ICU admission” refers to overall samples collected, regardless of if data were available for statistical analysis.

–, *P*-value not calculated; IQR, interquartile range; MDRO, multidrug resistant organism;

“n” represents the number of neonates for whom data were available and for whom statistical analysis was performed.

Further statistical analysis was not performed on the “day of life” variable, as 77/83 (93%) of the neonates were admitted within 24 h from birth to their respective sections, leading to comparable data with the “length of hospitalization” variable. Of note, 61/65 (91%) of MDRO colonized neonates were found to be colonized after 3 days of life (*P*-value = .0001).

Although 60.1% of MDRO colonized neonates had increased postnatal antimicrobial exposure (52/67 vs 13/16), this was not a statistically significant risk factor (*P*-value = .99). In fact, vancomycin exposure was significantly associated with the absence of MDRO colonization (*P*-value *=* .0028). On sub-analysis per MDRO type, however, carbapenem exposure was significantly associated with XDR *A. baumannii* colonization (10/19, *P-*value = .001).

MDR ESKAPE + *C. auris* isolation varied between the rectal, skin, and nasal swabs. Statistically, rectal and skin swabs performed similarly for the culture of MDR GNB, despite rectal swabs yielding the highest number of GNBs overall. All MRSA isolates were from nasal swabs, and all *C. auris* organisms were isolated from skin swabs (Table 4).

**Table 4.** Yield of various MDROs from colonization swabs

Organism	Rectal swabs (*n* = 86)		Skin swabs (*n* = 86)		Nasal swabs (*n* = 86)	
	MDRO	Total	MDRO	Total	MDRO	Total
*Acinetobacter baumannii*	MDR	0	MDR	0	MDR	2
	XDR	11	XDR	5	XDR	12
*C. auris*		0		2		0
*Enterobacter cloacae complex*	ESBL	3	ESBL	6	ESBL	4
	CRE	0	CRE	1	CRE	1
*Klebsiella pneumoniae*	ESBL	36	ESBL	38	ESBL	24
	CPE	2	CPE	0	CPE	0
	CRE	3	CRE	2	CRE	0
*Staphylococcus aureus*	MRSA	0	MRSA	0	MRSA	3
		55		54		46

CPE, carbepenemase-producing Enterobacterales; CRE, carbapenem-resistant Enterobacterales; ESBL, extended spectrum beta-lactamase; MDR, multidrug resistant; MDRO, multidrug resistant organism; MRSA, methicillin-resistant *Staphylococcus aureus*; XDR, extensively drug resistant.

## Discussion

Antimicrobial resistance affects various countries, but its impact is amplified in LMICs due to poverty, limited healthcare infrastructure and unequal access to resources which are commonly experienced in public hospitals in South Africa [27]. To our knowledge, this is the first study reporting on colonization of MDR ESKAPE + *C. auris* organisms in this region, despite infections from MDROs being associated with high mortality and increased healthcare costs [17, 28]. Previously published South African studies have focused on a limited pathogen spectrum [3, 13, 17]. Additionally, because standard microbiological methods compiled in SOPs were used, this study is reproducible using techniques available to researchers in diagnostic laboratories who would need to factor in the costs of additional consumables proportional to the number of samples collected.

In this study, over 80% of hospitalized neonates sampled were colonized with ESKAPE + *C. auris*, and the majority of these (97%) were MDR. The high burden of MDR ESKAPE + *C. auris* colonization in this unit is concerning. Moreover, 23% of neonates were colonized with multiple (≥2) MDROs. The high prevalence of colonization with MDR isolates in this study is consistent with published data on increasing clinical infections due to ESKAPE + *C. auris* isolates worldwide [4, 5, 13, 21]. However, inconsistent findings in the literature illustrate the uncertainty of the utility of colonization screening swabs in non-outbreak settings [16, 18, 29].

Multidrug-resistant GNB were predominant, commonly isolated from rectal and skin swabs. The predominance of ESBL *K. pneumoniae* mirrors data on invasive disease and colonization from the same unit and in other tertiary centers in South Africa [3, 5, 17]. Ogunbosi *et al*. [17] noted that 62.5% of ESBL-producing organisms isolated on screening swabs from children at a tertiary hospital in Cape Town, South Africa, were *K. pneumoniae* [17]. The low yield of CRE and CPE organisms was unexpected, but a study by Thomas *et al.* [4] details how carbapenem resistance was greater among *A. baumannii* compared to Enterobacterales from invasive sample cultures in the same unit, and Ogunbosi *et al*. reported a low CRE prevalence (0.5%) among their participants [17].

*A. baumannii* was the second most isolated organism and was principally carbapenem-resistant and XDR. This phenotype is a leading cause of neonatal infection and death in the same neonatal unit, accounting for 52% of infection-related deaths in a post-mortem study by Madhi *et al*. [28]. Carbapenemase-mediated resistance, solitarily NDM, accounted for a quarter of CRAB even among neonates without prior carbapenem exposure. Similarly, Nogbou *et al*. and Perovic *et al*. describe a prevalence of blaNDM possessing *A. baumanni* of 58% and 79%, respectively [30, 31]. This contradicts Lowe *et al*. who report that NDM-producing *A. baumannii* organisms occur to a lesser degree than OXA-type in South Africa [31].

Similar to previous authors, not all the risk factors assessed were found to be significant for MDRO colonization in this study [21, 32]. For instance, admission to the ICU regardless of duration, and most postnatal antibiotic exposures, were not found to be significant. Also, none of the neonates included within the first 24 h of life were colonized with MDR ESKAPE + *C. auris,* suggesting an unlikely role of vertical transmission in this group of patients. A longer duration of hospitalization was significantly associated with MDRO colonization in this study, a finding comparable to other authors [21, 32, 33]. This is partly attributable to the fact that hospitalized neonates have a lower species diversity of commensals, particularly when exposed to the hospital environment for prolonged periods [19]. Since adjusted and unadjusted logistic regression were not performed, risk factors found to be significant may represent underlying parameters that could not be defined individually. This includes vancomycin unexpectedly being associated with the absence of MDRO colonization. Due to the small number of patients in the study, this would require further exploration in a larger sample size.

*Staphylococcus aureus* and *E. faecium* were the only Gram-positive organisms investigated, explaining the low yield of GPCs (8.9%). This was further supported by data describing low neonatal *S. aureus* colonization at birth (2.5%), including neonates with maternal *S. aureus* colonization [20]. The absence of *P. aeruginosa* was anticipated, given how uncommon it is as a cause of neonatal sepsis [5, 7]. Although the prevalence of colistin resistance was 3/135 (0.05%), it is important to emphasize that colistin susceptibility was only performed on XDR *A. baumannii* isolates, and more recent data reports an increase in colistin resistance in South Africa [13, 34, 35].

### Strengths and limitations

There are several limitations to this study. Clinical data, such as gender, Apgar scores, the presence of an invasive device, and prematurity, were not assessed. Correlation with clinical infection before or following screening was not performed, nor was correlation with maternal characteristics, including prenatal antibiotic exposure and hospitalization. These factors and the sample size were greatly influenced by the objectives, funding, and laboratory processing in the primary investigation. This single-center study was cross-sectional, and thus, conclusions on the temporal progression of colonization could not be made, nor could any broader conclusions be drawn. It was also performed at a tertiary facility, and thus, findings may differ at other levels of care. Further characterization by whole-genome sequencing would offer more information on genotypic relatedness and the mechanism of colistin resistance, but could not be performed in the current study due to limited funding. With the above in mind, the study was able to describe reproducible and accessible microbiological methods relevant to an LMIC laboratory to identify specific colonization patterns that can inform diagnostic stewardship and infection control strategies.

Ascertaining the degree of colonization in neonatal units is an important preventative measure of horizontal transmission. Specific MDRO surveillance offers valuable information to identify colonized cases and inform the implementation of infection prevention and control measures. Identifying the anatomical site(s) with the greatest yield of the targeted MDRO can be valuable in minimizing cost and optimizing surveillance, in keeping with diagnostic stewardship and appropriate resource utilization. Molecular characterization to confirm the phenotypic characteristics documented in this study is required. In addition, further studies are required on the temporal characteristics of MDR colonization; to review the overall duration of colonization, individual risk factors associated with colonization, and to assess the long-term infective and non-infective consequences of MDR colonization. We hope to inspire readers in similar settings to strengthen surveillance systems in their units, which would assist in determining the prevalence of ESKAPE organisms and *C. auris* and emphasize preventative measures for healthcare.

## Supplementary Material

fmaf051_Supplementary_Data

## Data Availability

The authors confirm that the data supporting the findings of this study are available within the article [and/or its supplementary materials].
