# Supplementary material for: Prevalence of multidrug-resistant organisms colonizing neonates at a tertiary hospital in Johannesburg, South Africa
Source: J Trop Pediatr. 2026 Jan 2;72(1):fmaf051. doi: 10.1093/tropej/fmaf051 (PMC12758378; doi:10.1093/tropej/fmaf051)
Supplement: fmaf051_Supplementary_Data [file fmaf051_supplementary_data.zip › Mntla, supplementary material.pdf]

"Prevalence of multi-drug resistant organisms colonising neonates at a tertiary hospital in Johannesburg, South Africa", Mntla N, Chibabhai V and Nana T.

Supplementary Materials

Standard Operating Procedures

A: Standard operating procedure for the collection and processing of neonatal rectal swabs for the study: *Microbiological Surveillance of Hospital environmental surfaces, Medical equipment, Healthcare Workers, Patients and patients' parents in a tertiary hospital in Soweto, South Africa.*

1. **Purpose:**
    i. Provide detailed instruction on how to perform a rectal swab on neonates
    ii. Provide methodology for processing said clinical sample to identify patients colonized with carbapenem- resistant or carbapenemase – producing Enterobacterales/ Vancomycin Resistant Enterococcus faecium isolates in the intestinal tract
2. **Scope:**
   Performed by all competent staff trained in the collection and processing of clinical samples, this may include, but is not limited to: a CMID registrar, Medical technologist, Infection prevention and control personnel, and trained paediatric personnel.

3. **Responsibility:**
   All biomedical technologists, CMID registrars and IPC personnel involved in the collection of samples and processing.

4. **Definitions:**

   - CMID: Clinical Microbiology and Infectious Disease
   -CHBAH: Chris Hani Baragwanath Academic Hospital
   -CRE: Carbapenem Resistant Enterobacterale
   -VRE: Vancomycin resistant Enterococcus faecium
   -PPE: Personal protective equipment
   -IPC: Infection prevention and control
   -**ESKAPE-C pathogens**: Pathogens associated with nosocomial sepsis and drug resistance(1), namely:
    - *Enterococcus faecium*
    - *Staphylococcus aureus*
    - *Klebsiella pneumonia*
    - *Acinetobacter baumannii*
    - *Pseudomonas aeruginosa*
    - *Enterobacter species*
    - *Candida auris*

5. **Principle of procedure:**

A cross sectional qualitative description of the micro-organisms neonates at CHBAH are colonised/infected by, primarily aimed at selective recovery of CRE/VREs.(2)

6. Patient preparation and Specimen collection:

Specimens to be tested:

- Rectal swab only
    - No stool sample required

Transport requirement:

- To be placed in transport medium (Cary Blair medium); if possible, refrigerate Cary-Blair transport medium in advance, so that the swabs can be placed into a cool medium.

Patient Preparation:

- All swabs must be labelled prior to sampling

Rejection criteria:

- Un-labelled sample
- Sample incorrectly packaged
- Swab received beyond 24 hours after sampling and not placed on ice

7. Equipment and reagents:

	At Collection:	For Processing:
Equipment	PPE(non-sterile gloves and plastic apron)	Sterile scissors
	Rayon swabs (pre-moistened in Cary-Blair medium)	Sterile forceps
	Cary-Blair transport medium	Sterile loops 10µl
	Plastic sleeve	Bunsen burner
	Ice pack (maintain temp. 2-4∘C)	35∘C ± 2 aerobic incubator
	Cooler box	Vortex Mixer
		VITEK®-MS automated system
		VITEK® 2 automated system
Reagents and antimicrobials	Nil	Catalase reagent<br>Rapid indole<br>Oxidase
		Streptex kit
		5 ml Brain heart infusion broth(BHI)
		Vancomycin Etest<br>Teicoplanin Etest<br>Amphotericin B Etest

Agar plates	Nil	MacConkey agar plate (MAC)
		Cetrimide agar plate (C)
		Blood+ Nalidixic acid+ Colistin agar plates (BNC)
		Sabouraud dextrose agar (SDA)

8. **Environmental and safety controls**

   At specimen collection:
   - Restrict sampling to individual patient's cot
   - Attempt to avoid contamination of the swab while sampling by removing any obstructive items
   - Don appropriate PPE, which includes but is not limited to:

- Appropriately sized gloves
- Plastic apron

   - Seal the swab immediately after sampling in correct transport medium.

   At specimen processing:
   - A biological safety cabinet is not required for sampling of swabs
   - Material used for collection and or processing of samples is discarded into biohazard boxes lined with red plastic bags.
   - Needles and syringes should be disposed of into sharps containers.

9. **Quality Control**

Quality Control strains:	+	-
Catalase	*S. aureus* ATCC 25923	*Enterococcus faecalis ATCC 29212*
Aesculin bile agar/PYR	*Enterococcus faecalis ATCC 29212*	Streptococcus agalactiae ATCC10386
Rapid indole	*E. coli* ATCC 25922	*P. aeruginosa* ATCC 27853
Oxidase	*P. aeruginosa* ATCC 27853	*E. coli* ATCC 25922
Latex agglutination	*S. aureus* ATCC 25923	*Staphylococcus epidermidis ATCC 12228*

10. **Procedural steps: For Specimen Collection(2,3)**

(If possible, refrigerate Cary-Blair transport medium in advance, so that the swabs can be placed into a cool medium)

i. Wash hands and don appropriate PPE
ii. Expose the neonate
iii. Discard grossly contaminating stool with a wipe/ tissue paper
iv. Insert the premoistened rayon tipped swab 1-2cm into the anal canal
v. Rotate against rectal wall several times for 10 seconds

vi. Place the swab into transport media immediately, pushing it to the bottom of the tube.
vii. Tighten cap firmly. Transport swabs to the laboratory within the 24 hours after collection. Swab must be stored at 4 °C and below.

Procedure steps: For Swab processing (3–5)

i. Remove swab from packaging, and ensure correct swab (rectal) is selectedxs
ii. Remove polyester-tipped/rayon-tipped swab from Cary Blair transport medium
iii. Place immediately in 5ml BHI and vortex for 15 seconds(6)
iv. Incubate aerobically overnight and examine for turbidity at 24 and 48 hours.
v. Sub-culture turbid BHI using a 10µl loop onto: MAC+BNC+SDA+C. Streak out for single colonies
vi. Perform presumptive identification on colony morphology and rapid reagent testing
vii. Further identification of suspicious colonies to take place on Vitek-MS(BioMèriuux)
viii. If target organisms are identified antimicrobial susceptibility testing will take place on VITEK® 2 or appropriate manual methods as per CLSI standards.
ix. All processing is to be recorded in detail on the working cards provided and signed.
x. All working cards must be reviewed and signed by a CMID registrar.

Morphological features of target organisms:

- *Enterococcus faecium*: Small off-white to grey round colonies with α-haemolysis or Ƴ-haemolysis on 5% sheep blood agar.
- *Staphylococcus aureus*: Small round black colonies on Baird parker with a surrounding zone of clearing.
- *Klebsiella pneumonia*: Lactose fermenting on MacConkey agar, raised circular 2-3mm colonies with entire margins, often mucoid.
- *Acinetobacter baumannii*: Small 1-2mm round convex colonies with an entire margin that are non-lactose fermenting but light pink in colour and opaque on MacConkey agar.
- Pseudomonas aeruginosa: Non-lactose fermenting on MacConkey agar, flat spreading 2-3mm colonies with a wrinkled moist surface and a metallic sheen, oxidase Positive.
- *Enterobacter* spp: Grey to off-white spreading round colonies with entire to undulate margins on 5% blood agar.
- *Candida auris*: Large creamy whitish colonies on Sabouraud dextrose agar

	Lactose-fermenting (pink red) colonies	Perform rapid indole test	If **positiv**e= finalise as **non-ESKAPE** pathogen

MAC			If **negative**= continue to identify and perform susceptibility testing.
	Non-lactose fermenting colonies	Perform oxidase test	If **positive**, correlate with growth and pigmentation on CA plate_ presumptive *Pseudomonas aeruginosa*, but must confirm ID.
			Continue to ID **all** NLF isolates to species level, and only perform susceptibility testing on ESKAPE+ C organisms identified on the VITEK® 2 platform.
C (Correlate with growth on MAC)	Positive	Presence of growth on agar, with associated yellow-green to blue colour indicating the production of pyocyanin	Continue with ID, susceptibility only on *P. aeruginosa*
	Negative	No bacterial growth on agar plate	Finalise as non-ESKAPE pathogen
BNC	▪ **Streptococcal** morphology seen:	Perform Streptex	If agglutination for **group D** is present, suggests *Enterococcus spp.* Continue with ID an susceptibility from BNC Confirm Vancomycin and Teicoplanin MIC by Etest if resistant on VITEK® 2
			Agglutination with groups (A,B,C,F,G) or no reaction. Finalise

			as non-ESKAPE pathogen
	▪ **Staphylococcal** morphology seen	▪ Perform catalase test on similar sized colonies on MAC<br>▪ Perform Prolex latex Agglutination test	If **positive,** continue with ID. Perform susceptibility only on S. aureus isolates
			If **negative,** perform Streptex to exclude *Enterococcus* spp. If Negative, finalise as non-ESKAPE pathogen. If positive continue as mentioned above.
SDA	Assess for growth	Positive: White to cream coloured colonies	Continue with ID. Perform Amphotericin B E test only on *C. auris* isolates

References

1. Mulani MS, Kamble EE, Kumkar SN, Tawre MS, Pardesi KR. Emerging Strategies to Combat ESKAPE Pathogens in the Era of Antimicrobial Resistance: A Review. Front Microbiol [Internet]. 2019 Apr 1 [cited 2019 Dec 18];10. Available from: https://www.ncbi.nlm.nih.gov/pmc/articles/PMC6452778/
2. Richter SS, Marchaim D. Screening for carbapenem-resistant Enterobacteriaceae: Who, When, and How? Virulence. 2016 Nov 4;8(4):417–26.
3. Budding AE, Grasman ME, Eck A, Bogaards JA, Vandenbroucke-Grauls CMJE, van Bodegraven AA, et al. Rectal Swabs for Analysis of the Intestinal Microbiota. PLoS One [Internet]. 2014 Jul 14 [cited 2020 Jan 02];9(7). Available from: https://www.ncbi.nlm.nih.gov/pmc/articles/PMC4096398/
4. Kigbu A, Orimadegun AE, Tongo OO, Odaibo GN, Olaleye DO, Akinyinka OO. Intestinal Bacterial Colonization in the First 2 Weeks of Life of Nigerian Neonates Using Standard Culture Methods. Front Pediatr [Internet]. 2016 [cited 2020 Jan 02];4. Available from: https://www.frontiersin.org/articles/10.3389/fped.2016.00139/full
5. Manual of Clinical Microbiology, Twelfth Edition [Internet]. American Society of Microbiology; 2019 [cited 2020 Jan 02]. Available from: https://www.asmscience.org/content/book/10.1128/9781555819842
6. Liss MA, Nakamura KK, Peterson EM. Comparison of Broth Enhancement to Direct Plating for Screening of Rectal Cultures for Ciprofloxacin-Resistant Escherichia coli. Journal of Clinical Microbiology. 2013 Jan 1;51(1):249–52.

Compiled by Dr NM Mntla

<u>B: Standard operating procedure for the collection and processing of neonatal nasal swabs for the study: *Microbiological Surveillance of Hospital environmental surfaces, Medical equipment, Healthcare Workers, Patients and patients' parents in a tertiary hospital in Soweto, South Africa.*</u>

1. **Purpose:**
   i. Provide detailed instruction on how to perform a nasal swab on neonates
   ii. Provide methodology for processing abovementioned clinical sample to identify patients colonized with the ESKAPE + C pathogens.
2. **Scope:**
   Performed by all competent staff trained in the collection and processing of clinical samples, this may include, but is not limited to: a CMID registrar, Medical technologist, Infection prevention and control personnel, and trained paediatric personnel.

3. **Responsibility:**
   All biomedical technologists, CMID registrars and IPC personnel involved in the collection of samples and processing.

4. **Definitions:**
   - **CMID:** Clinical Microbiology and Infectious Disease
   - **CHBAH:** Chris Hani Baragwanath Academic Hospital
   - **PPE:** Personal protective equipment
   - **IPC:** Infection prevention and control
   - **MRSA:** Methicillin-resistant Staphylococcus aureus
   - **ET:** Endotracheal Tube
   - **ESKAPE+C pathogens**: Pathogens associated with nosocomial sepsis and drug resistance, namely(1):
   - *Enterococcus faecium*
   - *Staphylococcus aureus*
   - *Klebsiella pneumonia*
   - *Acinetobacter baumannii*
   - *Pseudomonas aeruginosa*
   - *Enterobacter species*
   - *Candida auris*

5. **Principle of procedure:**
   A cross sectional qualitative description of the multi-drug resistant organisms that neonates at CHBAH are colonised/infected by, primarily aimed at the selective recovery of MRSA(2) and other ESKAPE+ C organisms.

6. **Patient preparation and Specimen collection:**
   Specimens that may be used in this testing:
   Nasal swab only
   - In case of Nasal prong use: include swabbing over the surfaces of prongs from each nostril

- In case of intubation: include swabbing over ET and nasopharyngeal secretions

Transport requirement:

- To be placed in transport medium (Cary Blair medium); if possible, refrigerate Cary-Blair transport medium in advance, so that the swabs can be placed into a cool medium.

Patient Preparation:

- All swabs must be labelled prior to sampling

Rejection criteria:

- Un-labelled sample
- Sample incorrectly packaged
- Swab received beyond 24 hours after sampling and not placed on ice

7. Equipment and reagents:

	**At Collection:**	**For Processing:**
Equipment	PPE (non-sterile gloves and plastic apron)	Sterile scissors
	2mm Rayon swabs (pre-moistened in Cary-Blair medium)	Sterile forceps
	Cary-Blair transport medium	Sterile loops
	Plastic sleeve	Bunsen burner
	Ice pack (maintain temp. 2-4°C)	35°C ± 2C aerobic incubator
	Cooler box	Vortex Mixer
		VITEK® 2 automated system
		VITEK®-MS automated system
Reagents and antimicrobials	Nil	Catalase reagent<br>Rapid indole<br>Oxidase
		Streptex kit
		5 ml Brain heart infusion broth(BHI)
		Vancomycin Etest<br>Teicoplanin Etest<br>Amphotericin B Etest
Agar plates	Nil	Blood+ Nalidixic acid+ Colistin agar plates (BNC)
		Sabouraud dextrose agar (SDA)

		5% Sheep blood agar (BAP)
		Baird-Parker Agar (BP)
		MacConkey agar(MAC)
		Muller Hinton agar

8. Environmental and safety controls

At specimen collection:

- Restrict sampling to individual patient's cot
- Attempt to avoid contamination of the swab while sampling by removing any obstructive items
- Don appropriate PPE, which includes but is not limited to:

- Appropriately sized gloves
- Plastic apron

- Seal the swab immediately after sampling in correct transport medium.

At specimen processing:

- A biological safety cabinet is not required for sampling of swabs
- Material used for collection and or processing of samples is discarded into biohazard boxes lined with red plastic bags.
- Needles and syringes should be disposed of into sharps containers.

9. Quality Control

Quality Control strains:	+	-
Catalase	*S. aureus* ATCC 25923	*Enterococcus faecalis ATCC 29212*
Aesculin bile agar/PYR	*Enterococcus faecalis ATCC 29212*	Streptococcus agalactiae ATCC10386
Rapid indole	*E. coli* ATCC 25922	*P. aeruginosa* ATCC 27853
Oxidase	*P. aeruginosa* ATCC 27853	*E. coli* ATCC 25922
Prolex Latex agglutination	*S. aureus* ATCC 25923	*Staphylococcus epidermidis ATCC 12228*

10. Procedural steps: For Specimen Collection (3)

(If possible, refrigerate Cary-Blair transport medium in advance, so that the swabs can be placed into a cool medium)

i. Wash hands and don appropriate PPE
ii. Expose the neonate and rule out any nasal obstructions
iii. Insert a pre-moistened swab into each nostril at least 1cm deep or until resistance against the inferior turbinate is met (3)
iv. Rotate within the nostril and allow to remain in situ for at least 3 seconds to absorb

v. Using the same swab, repeat the process in the other nostril
    a. In the case of an infant on supplemental O2, include swab of nasal prongs
vi. Place the swab into transport media immediately, pushing it to the bottom of the tube.
vii. Tighten screw-cap firmly. Transport swabs to the laboratory within the 24 hours after collection, Swab must be stored at 4 °C and below.

Procedure steps: For Swab processing(4,5)

i. Remove swab from packaging, and ensure correct swab (nasal) is selected
ii. With sterile forceps, immediately place the nasal swab into the BHI liquid medium and vortex for 15 seconds
iii. Incubate overnight at 35 ± 2^{0}C under aerobic conditions (may require prolonged incubation if not turbid after 24 hours)
iv. Once solution has become turbid, vortex for 15 seconds.
v. Subculture using a 10µl loop onto: BAP+BNC+MAC+BP+SDA, and streak out for single colonies, incubate overnight at 35 ± 2^{0}C under aerobic conditions
vi. Perform presumptive identification on colony morphology and rapid reagent testing
vii. Further identification of suspicious colonies to take place on Vitek-MS(Biomerieux)
viii. If target organisms are identified antimicrobial susceptibility testing will take place on VITEK® 2 or appropriate manual methods as per CLSI standards.
ix. All processing is to be recorded in detail on the working cards provided and signed.
x. All working cards must be reviewed and signed by a CMID registrar.

Morphological features of target organisms:

- *Enterococcus faecium*: small off-white to grey round colonies with α-haemolysis or ϒ-haemolysis on 5% sheep blood agar.
- *Staphylococcus aureus*: Small round black colonies on Baird parker with a surrounding zone of clearing.
- *Klebsiella pneumonia*: Lactose fermenting on MacConkey agar, raised circular 2-3mm colonies with entire margins, often mucoid.
- *Acinetobacter baumannii*: Small 1-2mm round convex colonies with an entire margin that are non-lactose fermenting but light pink in colour and opaque on MacConkey agar.
- Pseudomonas aeruginosa: non-lactose fermenting on MacConkey agar, flat spreading 2-3mm colonies with a wrinkled moist surface and a metallic sheen, oxidase Positive.
- *Enterobacter* spp: Grey to off-white spreading round colonies with entire to undulate margins on 5% blood agar.
- *Candida auris*: Large creamy whitish colonies on Sabouraud dextrose agar

BAP	**Staphylococcal colony** morphology seen (Correlate with growth on Baird Parker Agar)	▪ Perform catalase test on similar sized colonies on MAC or Baird Parker Agar	If **positive,** continue with formal ID. Perform susceptibility only on S. aureus isolates
			If **negative,** perform Streptex to exclude *Enterococcus* spp (agglutination with group D reagent). If Negative, finalise as non-ESKAPE pathogen. If positive continue with ID and perform susceptibility only on *E. faecium* isolates
	**Streptococcal** morphology seen	Perform Streptex	If agglutination for **group D** is present, suggests *Enterococcus spp.* Continue with ID a susceptibility from BNC<br>Confirm Vancomycin and Teicoplanin MIC by Etest if resistant on VITEK® 2
			Agglutination with groups (A, B, C, F, G) or no reaction. Finalise as non-ESKAPE pathogen
	**Yeast-like colony** Morphology seen	▪ Perform a Gram to confirm	(See SDA)
	Lactose-fermenting (pink red) colonies	Perform rapid indole test	If **positiv**e= finalise as **non-ESKAPE** pathogen
			If **negative**= continue to identify and perform susceptibility testing.

MAC	Non-lactose fermenting colonies	Perform oxidase test	If **positive**, presumptive *Pseudomonas aeruginosa*, but must confirm ID.
			Continue to ID **all** NLF isolates to species level, and only perform susceptibility testing on ESKAPE+ C organisms identified
Baird Parker	Positive	Grey-black colonies with a halo ring	Continue with ID, susceptibility only on *S.aureus*
	Negative	No/other bacterial growth on plate	Finalise as non-ESKAPE pathogen
SDA	Assess for growth	Positive: White to cream coloured colonies	Continue with ID. Perform Amphotericin B E test only on *C.auris* isolates

References:

1. Mulani MS, Kamble EE, Kumkar SN, Tawre MS, Pardesi KR. Emerging Strategies to Combat ESKAPE Pathogens in the Era of Antimicrobial Resistance: A Review. Front Microbiol [Internet]. 2019 Apr 1 [cited 2019 Dec 18];10. Available from: https://www.ncbi.nlm.nih.gov/pmc/articles/PMC6452778/
2. Warnke P, Frickmann H, Ottl P, Podbielski A. Nasal Screening for MRSA: Different Swabs – Different Results! PLoS One [Internet]. 2014 Oct 29 [cited 2020 Jan 22];9(10). Available from: https://www.ncbi.nlm.nih.gov/pmc/articles/PMC4213029/
3. Frazee BW, Rodríguez-Hoces de la Guardia A, Alter H, Chen CG, Fuentes EL, Holzer AK, et al. Accuracy and Discomfort of Different Types of Intranasal Specimen Collection Methods for Molecular Influenza Testing in Emergency Department Patients. Annals of Emergency Medicine. 2018 Apr;71(4):509-517.e1.
4. Slingerland BCGC, Verkaik NJ, Klaassen CHW, Zandijk WHA, Reiss IKM, Vos MC. Neonatal Staphylococcus aureus acquisition at a tertiary intensive care unit. American Journal of Infection Control [Internet]. 2019 Dec 18 [cited 2020 Feb 21];0(0). Available from: https://www.ajicjournal.org/article/S0196-6553(19)30977-0/abstract
5. Manual of Clinical Microbiology, Twelfth Edition [Internet]. American Society of Microbiology; 2019 [cited 2020 Feb 22]. Available from: https://www.asmscience.org/content/book/10.1128/9781555819842

Compiled by Dr NM Mntla

C: Standard operating procedure for the collection and processing of neonatal skin (axillary/umbilical/groin)) swabs for the study: *Microbiological Surveillance of Hospital environmental surfaces, Medical equipment, Healthcare Workers, Patients and patients' parents in a tertiary hospital in Soweto, South Africa.*

1. **Purpose:**
   i. Provide detailed instruction on how to perform a screening skin swab on neonates
   ii. Provide methodology for processing the above-mentioned clinical sample to identify patients colonized with ESKAPE+C pathogens*.*
2. **Scope:**
   Performed by all competent staff trained in the collection and processing of clinical samples, this may include, but is not limited to: a CMID registrar, Medical technologist, Infection prevention and control personnel, and trained paediatric personnel.

3. **Responsibility:**
   All biomedical technologists, CMID registrars and IPC personnel involved in the collection of samples and processing.

4. **Definitions:**
   - **CMID:** Clinical Microbiology and Infectious Disease

   - **CHBAH:** Chris Hani Baragwanath Academic Hospital
   - **PPE:** Personal protective equipment
   - **IPC:** Infection prevention and control
   - **ESKAPE+C pathogens**: Pathogens associated with nosocomial sepsis and drug resistance, namely(1):
   - *Enterococcus faecium*
   - *Staphylococcus aureus*
   - *Klebsiella pneumonia*
   - *Acinetobacter baumannii*
   - *Pseudomonas aeruginosa*
   - *Enterobacter species*
   - *Candida auris*

5. **Principle of procedure:**

   A cross sectional qualitative description of the multi-drug resistant organisms neonates at CHBAH are colonised/infected by, primarily aimed at the selective recovery of *Candida auris, Pseudomonas aeruginosa* and *Acinetobacter baumannii* and other ESKAPE + C organisms
6. **Patient preparation and Specimen collection:**
   Specimens that may be used in this testing:
   - Skin swab only
     Areas of interest: Around the umbilicus, both axilla and the groin area(2)
   Transport requirement:

- To be placed in transport medium (Cary Blair medium); if possible, refrigerate Cary-Blair transport medium in advance, so that the swabs can be placed into a cool medium.

Patient Preparation:
- All swabs must be labelled prior to sampling
- Swab to be performed at least 48 hours after administration of topical antiseptic (e.g., chlorhexidine

Rejection criteria:
- Unlabelled sample
- Sample not in sterile packaging or contaminated
- Swab received beyond 24 hours after sampling, not placed on ice
- Non-patient specimen swabs.

7. Equipment and reagents:

	**At Collection:**	**For Processing:**
Equipment	PPE	Sterile scissors
	Standard Rayon swabs (pre-moistened in Cary-Blair medium)	Sterile forceps
	Cary-Blair transport medium	Sterile loops
	Plastic sleeve	Bunsen burner
	Ice pack (maintain temp. 2-4◦C)	35◦C ± 2◦C aerobic incubator
	Cooler box	Vortex Mixer
		VITEK®-MS automated system
		VITEK® 2 automated system
Reagents and antimicrobials	Nil	Catalase reagent<br>Rapid indole<br>Oxidase
		Streptex kit
		5 ml Brain heart infusion broth(BHI)
		Vancomycin Etest<br>Teicoplanin Etest<br>Amphotericin B Etest
Agar plates	Nil	5% sheep blood agar (BAP)
		MacConkey agar plate (MAC)
		Sabouraud Dextrose Agar (SDA)
		Cetrimide Agar (C)

8. Environmental and safety controls

At specimen collection:

- Restrict sampling to individual patient's cot
- Attempt to avoid contamination of the swab while sampling by removing any obstructive items
- Don appropriate PPE, which includes but is not limited to:

- Appropriate sized gloves
- Plastic apron

- Seal the swab immediately after sampling in correct transport medium to avoid contamination

At specimen processing:

- A biological safety cabinet is not required for processing of swabs; however, because *C. auris* may survive for weeks on plastic surfaces, with the potential to colonise healthy individuals, it is strongly advised that gloves, strong hand hygiene and decontamination with a disinfectant with sporociadal claim be used after work with *C. auris* cultures(3).
- Material used for collection and or processing of samples is discarded into biohazard boxes lined with red plastic bags.
- Needles and syringes should be disposed of into sharp's containers.

9. Quality Control

- All media and reagents in use should be examined for signs of contamination and expiration. Prior internal quality control testing will have to be conducted prior to use on clinical specimens to demonstrate growth or a positive reaction and to demonstrate inhibition or a negative reaction accordingly.
- All incubators in use need to have been recently serviced, and thermometers attached to observe the temperature.

Quality Control strains:	+	-
Catalase	*S. aureus* ATCC 25923	*Enterococcus faecalis ATCC 29212*
PYR	*Enterococcus faecalis ATCC 29212*	Streptococcus agalactiae ATCC10386
Latex agglutination test	*S. aureus* ATCC 25923	
Rapid indole	*E. coli* ATCC 25922	*P. aeruginosa* ATCC 27853
Oxidase	*P. aeruginosa* ATCC 27853	*E. coli* ATCC 25922

10. **Procedural steps: For Specimen Collection (2)**

(If possible, refrigerate Cary-Blair transport medium in advance, so that the swabs can be placed into a cool medium)

i. Wash hands and don appropriate PPE
ii. Expose the neonate
iii. Using a premoistened swab:
    a. Swab the umbilical stump in a linear direction, away from the stump opening
    b. Follow by swabbing with both the left axilla, and then right axilla concentrating on the creases, each for at least 3-5 times
    c. Using the same swab, rub across the left groin area, and then the right groin area, concentrating on the inguinal crease, swab in one direction at least 3-5 times
iv. Place the swab into Cary-blair transport media immediately
v. Transport swabs to the laboratory within the 24 hours after collection, Swab must be stored at 4 °C and below

Procedure steps: For Swab processing (2,4)

i. Remove swab from packaging, and ensure correct swab (skin) is selected
ii. With sterile forceps, Immediately place the skin swab into the BHI liquid medium and vortex for 15 seconds
iii. Incubate overnight at 35 ± 2^0C under aerobic conditions (may require prolonged incubation if not turbid after 24 hours)
iv. Once solution has become turbid, vortex for 15 seconds.
v. Subculture using a 10µl loop onto: BAP+C+MAC+SDA, and streak out for single colonies, incubate overnight at 35 ± 2^0C under aerobic conditions
vi. Perform presumptive identification on colony morphology and rapid reagent testing
vii. Further identification of suspicious colonies to take place on Vitek-MS(Biomerieux)
viii. If target organisms are identified antimicrobial susceptibility testing will take place on VITEK® 2 or appropriate manual methods as per CLSI standards.
ix. All processing is to be recorded in detail on the working cards provided and signed.
x. All working cards must be reviewed and signed by a CMID registrar.

Morphological features of target organisms:

- *Enterococcus faecium*: Small off-white to grey round colonies with α-haemolysis or Ƴ-haemolysis on 5% sheep blood agar.
- *Staphylococcus aureus*: Small round black colonies on Baird parker with a surrounding zone of clearing.
- *Klebsiella pneumonia*: Lactose fermenting on MacConkey agar, raised circular 2-3mm colonies with entire margins, often mucoid.

- *Acinetobacter baumannii*: Small 1-2mm round convex colonies with an entire margin that are non-lactose fermenting but light pink in colour and opaque on MacConkey agar.
- Pseudomonas aeruginosa: Non-lactose fermenting on MacConkey agar, flat spreading 2-3mm colonies with a wrinkled moist surface and a metallic sheen, oxidase Positive.
- *Enterobacter* spp: Grey to off-white spreading round colonies with entire to undulate margins on 5% blood agar.
- *Candida auris*: Large creamy whitish colonies on Sabouraud dextrose agar

<table>
<tr><td rowspan="5">BAP</td><td rowspan="2">Staphylococcal colony morphology seen</td><td rowspan="2">▪ Perform catalase test on similar sized colonies on MAC
▪ Perform a latex agglutination test</td><td>If positive, continue with formal ID. Perform susceptibility only on S. aureus isolates</td></tr>
<tr><td>If negative, perform PYR to exclude Enterococcus spp. If <u>Negative</u>, finalise as non-ESKAPE pathogen. If <u>positive</u> continue with ID and perform susceptibility only on E. faecium isolates</td></tr>
<tr><td rowspan="2">Streptococcal colony morphology seen</td><td rowspan="2">Perform Streptex</td><td>If agglutination for group D is present, suggests Enterococcus spp. Continue with ID a susceptibility from BNC
Confirm Vancomycin and Teicoplanin MIC by Etest if resistant on VITEK® 2</td></tr>
<tr><td>Agglutination with groups (A,B,C,F,G) or no reaction. Finalise as non-ESKAPE pathogen</td></tr>
<tr><td>Yeast-like colony Morphology seen</td><td>Perform a Gram to confirm</td><td>(See SDA)</td></tr>
</table>

MAC	Lactose-fermenting (pink red) colonies	Perform rapid indole test	If **positiv**e= finalise as **non-ESKAPE** pathogen
			If **negative**= continue to identify and perform susceptibility testing.
	Non-lactose fermenting colonies	Perform oxidase test	If **positive**, _ presumptive *Pseudomonas aeruginosa*, but must confirm ID.
			Continue to ID **all** NLF isolates to species level, and only perform susceptibility testing on ESKAPE+ C organisms identified on the VITEK® 2 platform.
SDA (correlate with BAP)	Assess for growth	Positive: White to cream coloured colonies	Continue with ID . Perform Amphoricin Etest on *C.auris* isolates only
C (Correlate with growth on MAC)	Positive	Presence of growth on agar, with associated yellow-green to blue colour	Continue with ID, susceptibility only on *P. aeruginosa*
	Negative	No bacterial growth on agar plate	Finalise as non-ESKAPE pathogen

References

1. Mulani MS, Kamble EE, Kumkar SN, Tawre MS, Pardesi KR. Emerging Strategies to Combat ESKAPE Pathogens in the Era of Antimicrobial Resistance: A Review. Front Microbiol [Internet]. 2019 Apr 1 [cited 2019 Dec 19];10. Available from: https://www.ncbi.nlm.nih.gov/pmc/articles/PMC6452778/
2. Screening for Candida auris Colonization | Candida auris | Fungal Diseases | CDC [Internet]. 2019 [cited 2020 Jan 23]. Available from: https://www.cdc.gov/fungal/candida-auris/c-auris-screening.html
3. Welsh RM, Bentz ML, Shams A, Houston H, Lyons A, Rose LJ, et al. Survival, Persistence, and Isolation of the Emerging Multidrug-Resistant Pathogenic Yeast Candida auris on a Plastic Health Care Surface. J Clin Microbiol. 2017 Oct;55(10):2996–3005.

4. Manual of Clinical Microbiology, Twelfth Edition [Internet]. American Society of Microbiology; 2019 [cited 2020 Feb 22]. Available from: https://www.asmscience.org/content/book/10.1128/9781555819842
